# Identifying sites where wild boars can consume anthropogenic food waste with implications for African swine fever

**DOI:** 10.1371/journal.pone.0308502

**Published:** 2024-08-08

**Authors:** Cecilia Aguilar-Vega, José Manuel Sánchez-Vizcaíno, Jaime Bosch

**Affiliations:** VISAVET Health Surveillance Centre and Animal Health Department, Complutense University of Madrid, Madrid, Spain; University of Naples Federico II: Universita degli Studi di Napoli Federico II, ITALY

## Abstract

Wild boar population dynamics promote the increase in numbers and distribution of the species in Eurasia, leading to a rise in the interaction with human activities, as well as generating problems with the management of certain infectious diseases, most notably African swine fever (ASF). ASF virus possesses high stability in several contaminated pork and pork products that can be a source of indirect transmission to susceptible hosts habituated to anthropogenic food waste. This transmission route is a concerning threat for the dispersion of the disease, primarily into unaffected areas given the worldwide widespread distribution of the disease and the increase of wild boar contact with humans. Thus, in this study, a straightforward tool to assess the relative risk of wild boar natural populations potentially consuming food waste is presented using synthetic data. Three risk groups were defined related to urban areas, travel, and leisure. The surrounding quality of habitat of wild boar was used to obtain the relative risk of wild boar potentially consuming anthropogenic food waste. To assign the relative risk to the corresponding risk unit, we also included the population for the urban areas group, and traffic volume for the travel risk group. The leisure group had higher scaled risk scores, followed by the urban areas group. Higher risk was found in the edges of the study area where more natural landscapes are found. The implications of this risk are discussed focusing on the context of ASF transmission. The outputs can help prioritize decision-making in terms of the improvement of preventive measures against the habituation of wild boar to anthropogenic food waste and ASFV introduction in a given study area.

## Introduction

Wild boar (*Sus scrofa*) populations are increasing in number in Europe [1]. In some regions of Asia, the abundance of wild boar was estimated to be higher than in Europe [2]. Overpopulation in some areas can cause loss of biodiversity, as well as dispersive movements to seek new sources of food. This overabundance can cause some conflicts with human activities, namely damage to crops and infrastructures, increase in traffic collisions with wildlife, increased transmission of infectious diseases to livestock and humans, and habituation of anthropogenic food waste consumption in accessible areas [1,3–5]. Wild boars are omnivores, being their diet mainly based on plants [4]. Differences in diet have been found depending on their habitat. Some wild boars prefer anthropogenic food (crops and garbage) depending on its accessibility and season [6–8]. One recent study showed that wild boars frequently visited parking areas for trucks in Finland, presumably to eat food waste [9]. Despite the different consequences of wild boar eating anthropogenic food waste, one important outcome of habituation to the ingestion of this type of food is the dissemination of diseases by contaminated food products by their etiological agents. This could be the case of African swine fever (ASF) [10,11].

ASF is a hemorrhagic disease that affects the Suidae family, and it has affected more than 50 countries outside its historically endemic region since 2007 [12,13]. Virulent strains can cause up to 90–100% mortality rates in highly susceptible species such as *Sus scrofa*, especially in domestic pigs [14]. The African swine fever virus (ASFV) has a high stability in organs, tissues and pork products (Tables 1 and 2). Hence, one concerning route of transmission is the ingestion by a susceptible host of ASFV-contaminated pork products. There are several introduction events of ASF linked to potentially contaminated pork products as well as food waste from airports and seaports, such as Portugal (1957), Brazil (1978), Malta (1978), Sardinia (1978), Belgium (1985), The Netherlands (1985), Georgia (2007), Sardinia (2023) [15–18]. The introduction of ASF in a free area has devastating consequences from an animal welfare and economic perspective. Economic impact derives from a decrease in pig population if domestic pigs are highly affected, but more importantly for exporting countries, due to export restrictions [19].

**Table 1:** African swine fever virus (ASFV) stability in organs and tissues of swine.

Organ/tissue	Ambient conditions	Last ASFV positive result	First ASFV negative result	Duration of the study	ASFV genotype (isolate)	ASFV detection method	Comment	Reference
Blood	4°C	75 weeks	-	75 weeks	Gt-I (Salamanca and Lisbon/60)	*in vitro*	Conserved in 0.5% EDTA	[20]
	Dark cold room	about 6 years	-	about 6 years	NS	*in vivo*	-	[21]
	-20°C	2 months	3 months	2 years	Gt-II (Estonia 2014)	*in vitro*	Data for domestic pig on an empty matrix	[22]
	4°C	3 months	6 months					
	RT	1 month	2 months					
Muscle	-20°C	2 years	-	2 years	Gt-II (Estonia 2014)	*in vitro*	Data for wild boar on an empty matrix. Domestic pig only tested on day 0	[22]
	4°C	1 month	2 months				Data for domestic pig on an empty matrix	
	RT	Day 0	1 week					
Skin	-20°C	Day 0	-	2 years	Gt-II (Estonia 2014)	*in vitro*	Data for domestic pig on an empty matrix	[22]
	4°C	Day 0	-					
	RT	1 month	2 months					
Bone marrow	-20°C	3 months	6 months	2 years	Gt-II (Estonia 2014)	*in vitro*	Data for domestic pig on an empty matrix	[22]
	4°C	1 month	2 months					
	RT	Day 0	1 week					
Spleen	-20°C	2 years	-	2 years	Gt-II (Estonia 2014)	*in vitro*	Data for domestic pig on an empty matrix	[22]
	4°C	1 week	1 month					
	RT	Day 0	1 week					
	-20°C	112 days	-	112 days	Gt-II (Pol16/20540/Out10)	*in vitro*	-	[23]
	4°C	56 days	112 days					
Kidney	-20°C	112 days	-	112 days	Gt-II (Pol16/20540/Out10)	*in vitro*	-	[23]
	4°C	28 days	56 days					
Lung	-20°C	112 days	-	112 days	Gt-II (Pol16/20540/Out10)	*in vitro*	-	[23]
	4°C	56 days	112 days					

Gt, genotype; RT, room temperature; NS, not specified.

^a^According to Kowalenko *et al*., 1965 [24] (as cited in [10,15]), ASFV can persist in muscle for 150 days at 4°C and 104 days at -4°C; additionally, it can persist in bone marrow for 6 months at -4°C [10,15].

**Table 2:** African swine fever virus (ASFV) stability in pork products.

Product	Ambient conditions	Last ASFV positive result	First ASFV negative result	Duration of the study	ASFV genotype (isolate)	ASFV detection method	Processing conditions	Comment	Reference
Canned brined ham	Cold room	ND	-	30 days	Gt-I	*in vitro* and *in vivo*	Hams were injected and submerged in a 16% brined solution at 4°C for 24 hours. Sealed cans were cooked in a 37.8°C water bath and slowly increased until the temperature of the ham was 69°C.	Viable ASFV was retrieved before the heating process 2 days after slaughter.	[25]
Pepperoni sausage	Dependent on processing conditions	8 days	30 days	60 days	Gt-I	*in vitro* and *in vivo*	Initially, several ingredients were mixed with ground meat. Curing: 4°C for 48 hours. Environmental control chamber: 20°C and 60% RH for 48 hours. Smoking chamber: 32.2 to 34.4°C and 85% RH for 8 hours. Drying: 11.7°C and 72% RH for at least 16 days.	*In vivo* studies performed 30 days after slaughter were negative. ASFV could not be retrieved after the required curing period.	[25]
Salami sausage	Dependent on processing conditions	9 days	30 days	60 days	Gt-I	*in vitro* and *in vivo*	Initially, several ingredients were mixed with ground meat. Curing: 4°C and for 48 hours. Environmental control chamber: 20°C and 68% RH for 48 hours. Smoking chamber 1: 32°C and 80% RH for 12 hours. Smoking chamber 2: 49°C and 58% RH for 12 hours. Drying: 11.7°C and 72% RH for at least 25 days.	*In vivo* studies performed 30 days after slaughter were negative. ASFV could not be retrieved after the required curing period.	[25]
Salami sausage	Dependent on processing conditions	18 days	26 days	137 days	Gt-I (Sardinia 49/04)	*in vitro* and *in vivo*	Initially, several ingredients were added. Cold room: 6°C for 12 hours. Drying 1: decreasing temperature from 22°C to 18°C and 70–99% RH for 3 days. Drying 2: decreasing temperature from 16°C to 12°C and 71–76% RH for 3 days. Maturing: 10–12°C and 75–80% RH.	Produced with 70% red ground meat and 30% fat. Commercial curing time: 27 days	[26]
Pork belly	Dependent on processing conditions	60 days	137 days	137 days	Gt-I (Sardinia 49/04)	*in vivo*		Commercial curing time: 14–21 days.	[26]
Loin	Dependent on processing conditions	83 days	137 days	137 days	Gt-I (Sardinia 49/04)	*in vivo*		Commercial curing time: 60 days.	[26]
Iberian loin	Dependent on processing conditions	98 days	112 days	140 days	Gt-I (INIA E-70)	*in vitro* and *in vivo*	-	Commercial curation time: 90–130 days.	[27,28]
Iberian ham	Dependent on processing conditions	112 days	140 days	196 days	Gt-I (INIA E-70)	*in vitro* and *in vivo*	-	Commercial curation time: 365–730 days.	[27,28]
Serrano ham	Dependent on processing conditions	112 days	140 days	168 days	Gt-I (INIA E-70)	*in vitro* and *in vivo*	-	Commercial curation time: 180–365 days.	[27,28]
Iberian shoulder ham	Dependent on processing conditions	112 days	140 days	196 days	Gt-I (INIA E-70)	*in vitro* and *in vivo*	-	Commercial curation time: 240–420 days for Iberian.	[27,28]
Parma Ham	Dependent on processing conditions	291 days	399 days	432 days	Gt-I	*in vivo*	Hams were salted. The process is thoroughly described in [29]. 0-4°C for 72 hours. Atmospheric chamber 1: 0-1°C and 75–85% RH for 28 days. Atmospheric chamber 2: 2.5–6.5°C and 65–80% for 62 days 15-18°C for 7 days. Curing: 15-20°C and 65–80% (reaching 85%) until the end of the curing phase.	At 291 samples were negative in the *in vitro* but positive in the *in vivo* experiment	[30]
Corned meat	22-25°C	16 days	-	60 days	Gt-II (Volgograd-Kalach 2012)	*in vitro*	Processed following a wet salting method.	-	[31]
	4-6°C	60 days	-	60 days					
	-16‒-20°C	60 days	-	60 days					
Canned meat	22-25°C	ND	Day 0	60 days	Gt-II (Volgograd-Kalach 2012)	*in vitro*	Prepared following the Russian Federation State Standards (GOST 32125–2013).	-	[31]
	4-6°C	ND	Day 0	60 days					
	-16‒-20°C	ND	Day 0	60 days					
Salted lard	4-6°C	ND	Day 0	60 days	Gt-II (Volgograd-Kalach 2012)	*in vitro*	Processed following dry salting method (OST 38–85 49).	-	[31]
	-16‒-20°C	60 days	-	60 days					
Pork sausage casings	Oscillating between 0-20°C and 40–90% RH	30 days	-	30 days	Gt-II (Georgia 2007/1)	*in vitro* and *in vivo*	-	Variant temperature and relative humidity using an environmental chamber.	[32]
	mean of 12.3°C (range: 0-26°C) and mean 74.1% RH (range: 20–100%)	30 days	-	30 days	Gt-II (Georgia 2007/1)	*in vitro*	-	Variant temperature and relative humidity using an environmental chamber.	[33]
	4°C	7 days	14 days	60 days	Gt-II (Georgia 2007/1)	*in vitro*	Samples incubated in medium with 10% an antibiotics mixture.	-	[34]

Gt, genotype; ND, not detected; RH, relative humidity.

Given the current distribution of ASF worldwide and the virus stability in pork and pork products, the transmission route by oral consumption of illegally introduced contaminated pork products is a concerning possibility in free areas of the disease [10,13]. Consequently, this study was focused on proposing an accessible and straightforward methodology to assess the relative risk of food waste consumption by wild boars in areas identified as potential sources, with implications for ASF dispersion in wildlife. This methodology can be applied without modification to the Eurasian region since it incorporates the quality of available habitat (QAH) for wild boar [35], although some assumptions of the model correspond to European Union legislation.

## Material and methods

### Identification of potential risk groups and their components (risk units)

Risk units were defined as areas potentially accessible to wild boar where anthropogenic food waste may be disposed of. Thus, food waste can be consumed by wildlife if not properly managed. These risk units were clustered in risk groups based on different activities or behaviors that lead to this indirect interaction with humans.

Three risk groups were identified and defined (Table 3). Urban areas can be an important feeding source for some wild boar populations. Food waste can be accessible in certain trash containers and public bins, and sometimes dwellers actively feed wildlife [36]. Another risk group is related to land transportation and road travel. In this scenario, long-distance travelers from different geographical regions can stop in gas stations to fuel their vehicles and rest, or in rest areas with the later purpose. Some travelers can eat food from their origin region and leave the food waste there, accessible to wild boars [9]. The last identified risk group includes leisure activities in or close to natural landscapes. These activities can be carried out inside or around protected natural areas, that attract national and international tourism.

**Table 3:** Definition of the risk groups identified by human activity included in the study.

Risk group	Risk units	Type of data
Urban areas	Urban areas	Point shapefile
Travel	Gas stations and rest areas	Point shapefile
Leisure activities	Parking, camping, picnic areas, recreational areas, and viewpoints, inside or near protected natural areas as well as close to natural landscapes	Point shapefile

### Quantification of the risk of wild boar eating food waste in the risk units within the defined risk groups

For the purpose of the study synthetic data was generated (S1 Appendix) in a study domain of two latitudinal and three longitudinal decimal degrees. To assess the risk of wild boar eating food waste, firstly, the population at risk needed to be defined. The quality of available habitat (QAH) for wild boar was used as a representation of where wild boars most probably inhabit in greater numbers [35]. In general, QAH 1.75 and 2 are the QAH categories more favorable for wild boar populations [35]. A 2 km-radius buffer around risk units was generated, and the percentage of high QAH, expressed on a scale of 0–1, was used as an estimate of the relative risk of wild boar potentially consuming food waste (hereafter wild boar risk indicator -*R*) in each risk unit.

In the case of urban areas, polygon data can be used. If so, we recommend when doing the buffer to exclude the polygon urban area for the analysis. For this risk group, we assumed that higher numbers of human population represented a higher volume of food waste, and therefore, a higher potential risk of wild boar eating food waste. Given the wide range of populations that can be present in different administrative units of a given region [37], the number of dwellers per administrative unit (*P*_*i*_) was transformed to *log*_10_(*P*_*i*_). The wild boar risk indicator for each risk urban unit (*R*_*ui*_) was then calculated (Eq 1).

$$R_{ui}=q_{wbi}\cdot {log}_{10}(P_{i})$$ (1)

where *q*_*wbi*_ is the proportion of QAH of categories 1.75 and 2 in the buffer around each urban unit, and *P*_*i*_ is the number of dwellers per administrative unit *i*.

For travel units, traffic on highways, expressed as the mean number of vehicles, was considered as an estimator of the amount of food waste from travelers that could be available at each risk unit. Only nearby risk units, within 800 meters of a road with traffic information, were considered at risk. The reasoning behind it is that risk units associated with land transport outside of the main highways and with less traffic will have less influx of transient travelers, and therefore, less or even negligible risk of accessible food waste from this route. The mean traffic (*MT*) for each risk unit related to travel *j* was transformed to *log*_10_(*MT*_*j*_ + 1). The wild boar risk indicator for each travel unit (*R*_*tj*_) was then calculated (Eq 2).

$$R_{tj}=q_{wbj}\cdot {log}_{10}({MT}_{j}+1)$$ (2)

where *q*_*wbj*_ is the proportion of QAH of categories 1.75 and 2 in the buffer around each travel risk unit, and *MT*_*j*_ is the mean intensity of traffic for each risk unit *j*.

For the risk group of leisure activities, the wild boar risk indicator (*R*_*rk*_) was exclusively associated with the proportion of QAH of categories 1.75 and 2 in the buffer around each leisure unit *k* (Eq 3).


$$R_{rk}=q_{wbk}$$ (3)

The wild boar risk indicator for each risk unit in the urban and travel risk groups was scaled to a range between 0 and 1. The non-parametric Kruskal–Wallis test was applied to statistically assess differences in the wild boar risk indicators between groups (urban, travel, and leisure), considering a significance level of *p* < 0.05.

Each risk group was aggregated by the administrative unit for the graphical representation. For the urban areas risk group, the scaled wild boar risk indicator was assigned to its corresponding administrative unit. Whilst, for the travel and recreational activities risk groups the sum of every risk unit contained in each administrative unit was used.

Geospatial and statistical analyses were developed in ArcGIS Pro v.2.9, as well as R v.4.2.2 [38], using the following packages for data manipulation (“dplyr” [39], “sf” [40], “raster” [41]), and graphical representation (“ggplot2” [42], and “gridExtra” [43]).

## Results

**Fig 1:**
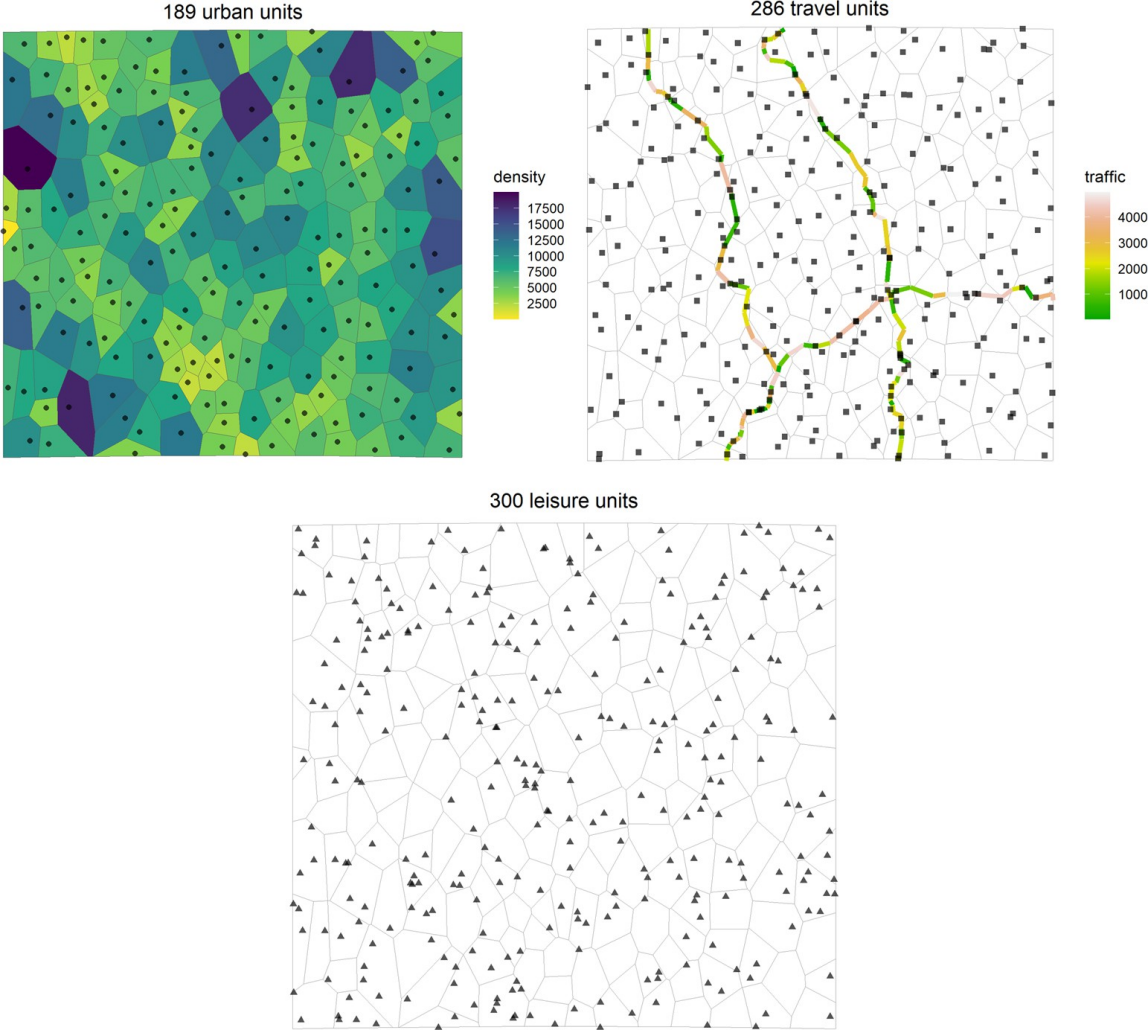
Distribution of the three risk groups: Urban areas, travel, and leisure. The image was generated in R v.4.2.2. In the urban map, the human population is represented per administrative unit (in number of dwellers). In the travel map, the traffic is represented (mean number of vehicles).

**Fig 2:**
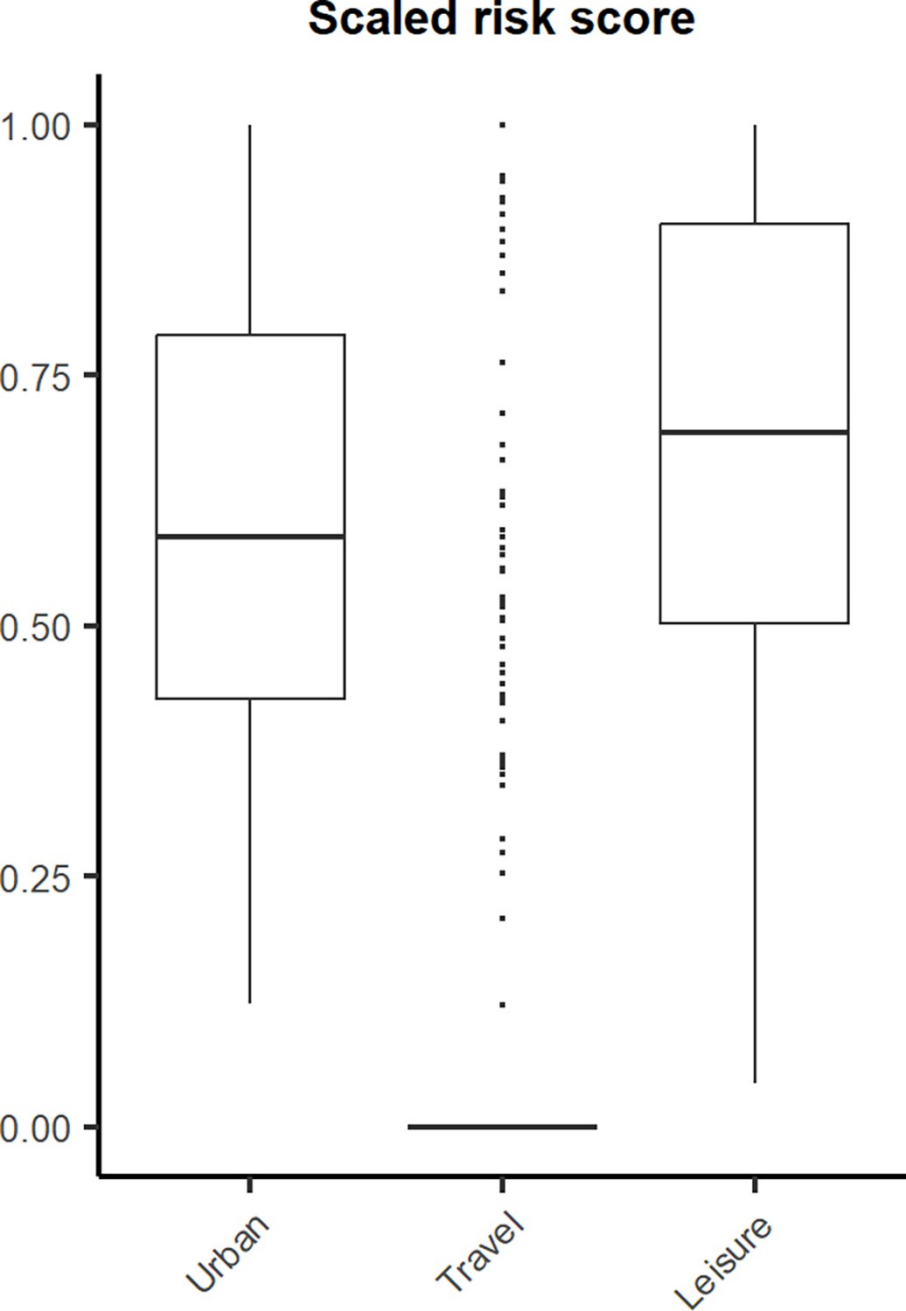
Scaled wild boar risk indicator for each risk group included in the study. The image was generated in R v.4.2.2.

A total of 775 synthetic risk units were generated for the study area distributed in 189 urban units (one per administrative unit), 286 travel units, and 300 leisure units (Fig 1). The leisure risk group had a greater wild boar risk indicator, followed by the urban areas risk group (Fig 2). Statistical analysis showed significant differences in wild boar risk indicators for the different groups (*Χ*^*2*^ = 411.78, *df* = 2, *p* < 0.05).

Leisure units were located in areas with more quality and abundance for wild boar, with a mean of 68.3% 95% CI [65.59, 71.01] for high QAH (1.75 and 2), closely followed by urban areas (66.48% 95% CI [63.21, 69.76]) and travel units (65.07% 95% CI [62.32, 67.81]). However, the travel risk group had lower risk values due to the high distance (>800 meters) to roads with intense relevant traffic from 233 travel units.

**Fig 3:**
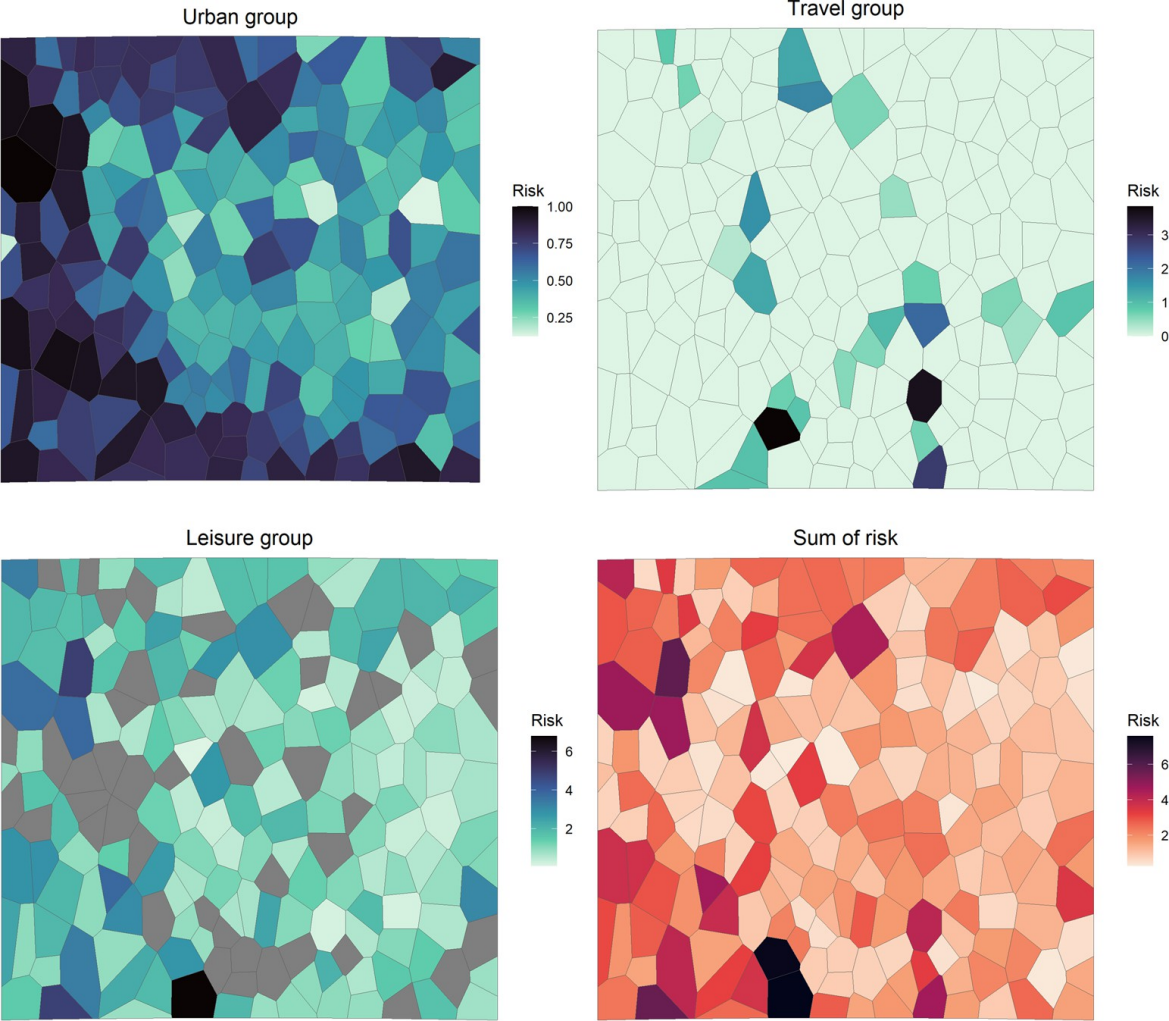
Relative risks for the three risk groups by administrative unit (urban areas, travel and leisure units), and aggregation of relative risks of wild boars eating food waste in the study area. Gray areas represent administrative units with no leisure units. The image was generated in R v.4.2.2.

Fig 3 shows the wild boar risk indicator (*R*) for each risk group at the administrative unit level. Moreover, the final wild boar risk indicator map shows administrative units where more risk of indirect interaction of natural populations of wild boar with humans can occur. The urban areas with more risk were located primarily on the north, east, and south edges of the study area where the majority of land cover is forest and semi-natural areas [44]. Similarly, the relative risk of leisure units was more abundant in natural areas. The wild boar risk indicator for travel units was highly influenced by the presence of highways, as modeled.

## Discussion

In this study, we described a methodology to spatially assess the relative risk of wild boar eating anthropogenic food waste. This human-wildlife contact can favor, among other issues, the bidirectional transmission of zoonotic and non-zoonotic diseases of the human-wildlife interface [3]. In the case of ASF, ASFV-contaminated food waste can be a source of infection for susceptible hosts [15,16]. This methodology can therefore be applied in areas free of a certain disease (such as ASF), to evaluate and improve prevention measures, as well as in already affected areas to avoid reintroductions and the onset of new cases, or even new ASFV strains. Although the methodology could be applied to the extension of the QAH raster [35], which is the Eurasian region, it is worth noting that legislation and sociocultural differences can also influence this type of study. For example, domestic pig populations were excluded from this study since swill feeding is prohibited in the European Union in accordance with Regulation (EC) No 1069/2009; however, in other countries from the Eurasian region, it is a common practice [45,46]. Furthermore, in some areas, landfills can be more accessible to wildlife. On the other hand, in insular settings with strong customs at seaports and airports, the travel risk group could be deemed negligible; although some studies that will be discussed later should be taken into consideration. Thus, the inclusion of other risk groups or exclusion of the ones presented here should be considered when preparing the application of the methodology to a specific location, always reflecting the specific characteristics of a given study area.

Although this analysis provides a general tool for the wild boar risk indicator in each risk unit, seasonality should be considered in the interpretation of the results. Firstly, the abundance and behavior of wild boar vary depending on the season due to food availability and hunting seasons [47,48]. Climatic conditions modify the foraging and eating behavior of wildlife due to the seasonality of natural resources [47]. When there is less availability of natural resources, habituated wild boars are more prone to seek anthropogenic food and water sources [6,49,50]. In addition, the visits by humans to leisure areas located in natural landscapes are conditioned by climate, being often more concurred when temperatures are mild and with a lack of intense precipitation.

The individual and final wild boar risk indicator maps (Fig 3) show administrative units where more risk of indirect interaction of natural populations of wild boar with humans can occur. Risk groups would have differences in ASF risk of introduction. The travel risk group is the most comprehensible one. International travelers may bring food from their original destination (that may be an ASF-affected region) and throw potentially ASF-contaminated food waste in travel risk units [9,51]. In ASF-free areas, urban and leisure risk groups can contribute to the risk of ASF introduction into wildlife populations due to dwellers with familiar, work, or other connections with ASF-affected regions. In these scenarios, they could illegally bring traditional pork products originating from activities with lesser sanitary control such as familiar pig slaughtering or hunting from affected regions. One of the pillars of the EU is the free movement of people and goods, and several studies concluded a non-negligible risk of illegal importation of pork products to ASF-free countries at airport customs [52,53]. As an addition to this type of study, a risk assessment of the introduction of ASF could be included for the study area and combined with the risk of exposure of wildlife. To properly assess the risk of introduction of ASF in the area of study several parameters should be considered. Dwellers and tourists with connections to ASF-affected areas should be quantified and weighed according to the epidemiological situation of those areas.

Nevertheless, the methodology provided here can be valuable to prioritize the application of measures focused on minimizing wild boar habituation to anthropogenic food waste and the management and sanitary consequences it implies. Proper awareness and management of food waste is essential to achieve this goal. The type of trash and bin containers is an important factor in the accessibility of food waste to wildlife [54]. The number and characteristics of trash containers were not included since usually, this information is rarely available or obtainable for large study areas. Ideally, the location of trash bins and garbage containers should be used for the analysis including factors that may affect the accessibility to wildlife. Underground containers and wildlife-proof trash containers are the optimal methods to hamper wildlife accessibility to food waste [54]. For urban areas, underground containers are an efficacious measure. However, the inappropriate use of some of these services by the population may nullify the applicability of their design. Such would be the case if someone were to throw garbage outside the container or leave open the gate of a fenced area where garbage containers are confined. To minimize the impact of uncivil behavior two complementary courses of action can be applied. The first one would be to raise people’s awareness about their sanitary implications using informational signage [51]. The other would be removing the garbage before nighttime when wild boars are usually more active [49,55]. The latter measure would be especially effective for the leisure risk group because they are visited mainly during the day, except for camping areas. In combination with these measures, the reduction of wild boar overabundance would be highly beneficial for biodiversity, reduce conflicts with humans, and minimize the spread and impact of infectious diseases [3,51,56].

### Conclusion

In this study, we presented a methodology that can be applicable to areas in a large geographical region where there is a wide distribution of wild boar. It allows to identify areas with more risk of indirect interaction of natural populations of wild boar with humans in terms of eating anthropogenic food waste. These findings can be beneficial to enhance wild boar management and cost-effective preventive measures against the habituation of wild boar consuming anthropogenic food waste and ASFV introduction or re-introductions in areas of the Eurasian region.

## Supporting information

S1 Appendix Generation of synthetic data. (DOCX)
