# Supplementary material for: Identifying sites where wild boars can consume anthropogenic food waste with implications for African swine fever
Source: PLoS One. 2024 Aug 8;19(8):e0308502. doi: 10.1371/journal.pone.0308502 (PMC11309469; doi:10.1371/journal.pone.0308502)
Supplement: S1 Appendix — (DOCX) [file pone.0308502.s001.docx]

Identifying sites where wild boars can consume anthropogenic food waste with implications for African swine fever

**S1 Appendix**

**Generation of synthetic data**

The study domain was generated to have two decimal degrees latitude and three decimal degrees longitude. This process was performed in R v.4.2.2 [1], using the following packages: “dplyr” [2], “sf” [3], “raster” [4], “ggplot2” [5], and “gridExtra” [6].

**Urban areas and administrative units**

A set of 300 random points were generated within the study domain. Subsequently, some points were removed so the minimum distance between points was 8 km. The remaining points were considered as the synthetic urban areas.

Administrative units were generated from the urban risk points performing a Voronoi tessellation.

The population of each urban risk unit was estimated based on the area of each administrative unit polygon, and ranged between 20 and 20,000. A min-max scaling was performed as follows:

$$x^{'}=a+\frac{\left( x-\min\left( x \right) \right)\cdot(b-a)}{\max\left( x \right)-min(x)}$$

being: $x^{'}$: the new estimated population density

$x$: the area of the administrative unit polygon

$a$: the minimum population (20 habitants)

$b$: the maximum population (20,000 habitants)

**Travel risk units**

Usually, there are gas stations and rest areas in close proximity to highways, accessible to travelers. In addition, gas stations provide service to dwellers. Hence, two approaches were followed to recreate the disposition of such structures.

For travel risk units associated with highways, the first step was generating the highways using points that were transformed into polylines. Subsequently, a buffer of 200 m radius was created around them in ArcGIS Pro v.2.9. To assign traffic (mean number of vehicles) to the highways, the polylines were segmented by vertices in the same program. Then, random integer values (up to 5,000) were produced and assigned to each segment in R. Within the road buffer, 50 random points were generated.

For the other gas stations, at least one unit was randomly created in each administrative unit; another unit was randomly created in administrative units with an area in the fourth quartile.

**Leisure risk units**

Leisure units are usually located in or close to areas of natural interest and pintoresque landscape. This kind of landscape also corresponds to more natural areas. Thus, the values of the quality of available habitat (QAH) for wild boar [7], was used as probability weights to generate random points.

**References**

1. R Core Team. R: A Language and Environment for Statistical Computing. Vienna, Austria: R Foundation for Statistical Computing; 2022.

2. Wickham H, François R, Henry L, Müller K. dplyr: A Grammar of Data Manipulation. R package version 1.0.7 2021. Available from: <https://CRAN.R-project.org/package=dplyr>.

3. Pebesma E. Simple Features for R: Standardized Support for Spatial Vector Data. The R Journal. 2018;10(1):439-46. doi: 10.32614/RJ-2018-009.

4. Hijmans RJ. raster: Geographic Data Analysis and Modeling. R package version 3.6-14 2023. Available from: <https://CRAN.R-project.org/package=raster>.

5. Wickham H. Elegant Graphics for Data Analysis 2016. Available from: <https://ggplot2.tidyverse.org>.

6. Auguie B. gridExtra: Miscellaneous Functions for "Grid" Graphics. R package version 2.3. 2017. Available from: <https://CRAN.R-project.org/package=gridExtra>.

7. Bosch J, Iglesias I, Muñoz MJ, de la Torre A. A Cartographic Tool for Managing African Swine Fever in Eurasia: Mapping Wild Boar Distribution Based on the Quality of Available Habitats. Transbound Emerg Dis. 2017;64(6):1720-33. doi: 10.1111/tbed.12559.
